# An Interesting Recital

**Published:** 1921-03-12

**Authors:** 


### An Interesting Recital.

Members of the College of Nursing who attended the lectures on "Public Speaking" which Miss Louie Bagley gave will be interested to learn that she is giving a Recital at the Steinway Hall on April 7, at 8.15 p.m. Miss Bagley is recognised as an able trainer for public work of all kinds. She holds that the art of using the beautiful sounds of the English language in speech is a great one, and that it is an immense and largely undeveloped power in modern days. The Recital on April 7 of Poetry, Prose, and Drama will develop this theme. Miss Bagley will be assisted by Mr. Ernest Bonner on the violoncello, and Mr. A. Thompson and Mrs. Bonner on the organ and pianoforte.

